# Nutritional and Exercise-Focused Lifestyle Interventions and Glycemic Control in Women with Diabetes in Pregnancy: A Systematic Review and Meta-Analysis of Randomized Clinical Trials

**DOI:** 10.3390/nu15020323

**Published:** 2023-01-09

**Authors:** Cassy F. Dingena, Daria Arofikina, Matthew D. Campbell, Melvin J. Holmes, Eleanor M. Scott, Michael A. Zulyniak

**Affiliations:** 1 Nutritional Epidemiology Group, School of Food Science and Nutrition, University of Leeds, Leeds LS2 9JT, UK; 2 School of Nursing and Health Sciences, Institute of Health Sciences and Wellbeing, University of Sunderland, Sunderland SR1 3SD, UK; 3 Division of Clinical and Population Sciences, Leeds Institute of Cardiovascular and Metabolic Medicine, University of Leeds, Leeds LS2 9JT, UK

**Keywords:** maternal diabetes, diet, nutritional supplementation, physical activity, randomized controlled trials, glucose control, diabetes management

## Abstract

Diabetes disrupts one in six pregnancies, bestowing immediate and long-term health risks to mother and child. Diet and exercise are commonly prescribed to control dysglycemia, but their effectiveness across sub-populations and types of diabetes (type-1; type-2; or gestational diabetes mellitus, GDM) is uncertain. Therefore, a systematic review and meta-analysis on the effect of diet and/or exercise on glycemia in pregnant women with diabetes was conducted. Random effects models were used to evaluate effect sizes across studies and anticipated confounders (e.g., age, ethnicity, BMI). Of the 4845 records retrieved, 26 studies (8 nutritional supplements, 12 dietary, and 6 exercise interventions) were included. All studies were conducted in patients with GDM. Overall, supplement- and exercise-based interventions reduced fasting glucose (−0.30 mmol/L; 95% CI = −0.55, −0.06; *p* = 0.02; and 0.10 mmol/L; 95% CI = −0.20, −0.01; *p* = 0.04); and supplement- and diet-based interventions reduced HOMA-IR (−0.40; 95% CI = −0.58, −0.22; *p* < 0.001; and −1.15; 95% CI = −2.12, −0.17; *p* = 0.02). Subgroup analysis by confounders only confirmed marginal changed effect sizes. Our results suggest a favorable role of certain nutritional supplements, diet, and exercise practices on glycemia in women with GDM and underline a lack of evidence in ~20% of other diabetes-related pregnancies (i.e., women with pre-existing diabetes).

## 1. Introduction

Diabetes in pregnancy (DIP) is one of the most common complications during pregnancy, with 16.7% of live births (in 2021) being affected by diabetes [1]. DIP is classified by the development of diabetes during pregnancy (i.e., gestational diabetes mellitus, GDM) or by women diagnosed with type 1 or type 2 diabetes before becoming pregnant (T1D or T2D, respectively), of which GDM comprises 80% of all cases of DIP [1]. Women with DIP are at a 3-fold higher risk of adverse maternal and infant pregnancy outcomes and are at long-term risk of comorbidities compared to women without DIP [2]. Adverse pregnancy outcomes include fetal macrosomia, stillbirth, neonatal metabolic disturbances, preeclampsia, and cesarean delivery [3,4,5]. Furthermore, women with DIP are at risk of developing T2D, while their offspring are at increased risk of early-life glucose intolerance and obesity in later life [3,6]. These adverse intrauterine environmental exposures are hypothesized to introduce epigenetic modifications to the fetus that contributes to metabolic disorders throughout life and future generations [6,7].

All women diagnosed with DIP require antenatal care to minimize short- and long-term complications. Glycemic control may be achieved by a combination of diet, weight management, exercise, blood glucose monitoring, and pharmacologic treatments (e.g., metformin or insulin) [1,8,9]. In the UK, pregnant women with any form of diabetes are advised to aim for plasma glucose below the following target levels—fasting: 5.3 mmol/L and 1 h post meals: 7.8 mmol/L or 2 h post meals: 6.4 mmol/L—according to National Institute for Health Care Excellence (NICE) [9]. Key strategies to achieve these targets are embedded in the promotion of pregnancy lifestyle habits that include a healthy diet (e.g., whole grains, fruits, and vegetables) and regular physical activity. Such guidelines can be highly effective and contribute to the healthy management of DIP in 70–85% of women with DIP [9,10]. The NICE guidelines primarily focus on improving carbohydrate quality [by including lower glycemic index (GI) foods] and physical activity habits to manage glycemia during pregnancy [9]. However, while numerous studies support the prescription of balanced diets for the management of mean glucose levels, their effect on reducing episodes of hypo- and hyperglycemia and ability to reduce maternal and offspring risk of complications is not clearly established with recent work highlighting significant heterogeneity in their effectiveness [11,12,13]. Additionally, most studies do not consider physical activity, which can interact with and modify the effect of diet on glycemic control and the health of the mother and offspring [3,14,15,16]. In short, an investigation into the generalizability of evidence and key lifestyle moderators (i.e., diet and/or exercise) of dysglycemia in pregnancy is needed.

Growing research with established glucose measures and continuous glucose monitors (CGM) has shed light on numerous lifestyle-dysglycemia associations and novel points of interest for managing dysglycemia during pregnancy and its associated health risks [11,12,17,18]; however, emerging research postulates women with DIP and their offspring remain at risk [17,19,20,21]. This systemic review and meta-analysis aimed to investigate the magnitude and generalizability of the effects of nutritional supplements, diet, and/or exercise on glycemic control in women with DIP.

## 2. Materials and Methods

The guidelines of Preferred Reporting Items for Systematic Reviews and Meta-Analyses (PRISMA) were followed for conducting this systematic review and meta-analysis [22]. This study was registered with PROSPERO (CRD42021268977). This review aimed to investigate the following question:

Do diet and/or exercise interventions improve maternal glucose (fasting and postprandial glucose levels, glycated hemoglobin levels, and insulin resistance) in women diagnosed with DIP when compared to the control intervention?

### 2.1. Search Strategy and Study Selection

Cochrane, AMED, EMBASE, MEDLINE (via OVID), PubMed, and Scopus were searched to identify randomized controlled trials (RCTs) relevant to the ‘lifestyle’ interventions and glycemia in DIP. Full search terms are presented in Supplemental Materials Table S1. Additional manual searches were conducted by reviewing reference lists of included articles and relevant reviews.

The screening was performed in duplicate and independently by two authors, first by reviewing titles and abstracts and then by reviewing the full texts to identify all eligible RCTs articles. Included studies were randomized controlled trials and crossover studies, either acute (assessing single meal response/intake < 2 weeks) or long-term (assessing intake > 2 weeks), investigating the effect of diet and/or exercise interventions in comparison with control on parameters of glycemic control measured using capillary or venous blood in women diagnosed with DIP (T1D, T2D, or GDM). Studies were excluded if they did not report diet and/or exercise interventions, were focused on children and adolescents (<18 years of age) or women >45 years of age with comorbidities (e.g., cardiovascular disease and cancer, etc.), or if the outcome measures of glycemic control were not reported. The trials included were limited to being published after the year 2000, and peer-reviewed RCTs or crossover studies were available as full texts in English. Corresponding authors were contacted to request the full text where articles were not accessible online.

### 2.2. Data Extraction and Quality Assessment

The following data were extracted from included studies: first author and year of publication; publishing journal; country of study; sample and estimated power of sample size; definition of GDM diagnosis used; design of the study (RCT vs. crossover study); intervention and control (type, dose, and format of intervention); study duration and participant characteristics (age, body mass index (pre-pregnancy or at enrolment), weeks of gestation at enrolment); primary/secondary outcomes. The outcome measures of included studies were extracted as means and its variance (e.g., mean difference (MD), standard deviation (SD), standard error (SE), confidence interval (CI), etc.) of baseline and post-intervention fasting plasma glucose (FPG; mmol/L), post-prandial glucose (PPG; mmol/L), glycated hemoglobin (HbA1c; %), and insulin resistance expressed as Homeostatic Model of Assessment (HOMA-IR). In cases where data were presented in alternative units (e.g., mg/dL), they were converted to mmol/L. The following formula was used: total glucose in mg/dL divided by 18.0182 mmol L^−1^/1 mg dL^−1^. If data were presented in figure format, values were extracted using Web Plot Digitizer [23].

Bias assessment of the individual studies was conducted using the updated Cochrane Collaboration tool for assessing the risk of bias (RoB2) [24]. The studies were categorized into three categories—high risk, low risk, or some concerns raised—in six domains, which are as followed: randomization process, deviations from intended interventions, missing outcome data, measurement of the outcome, selection of the reported results, and overall bias. The tool uses an algorithm based on signaling questions to assess the risk of bias for each domain as well as provide an overall risk of bias assessment. Publication bias was assessed by visual inspection of funnel plots.

### 2.3. Data Analysis

Data were analyzed using Review Manager (RevMan; version 5.4.1; The Cochrane Collaboration, 2020). Trials not reporting uncertainty of effect sizes (e.g., standard deviation, standard error, or confidence interval) were excluded from the meta-analysis. Pooled, weighed, fixed, and random effects analyses were performed to estimate the mean difference of effect (MD) of nutritional supplement-, dietary-, or exercise-based trials on DIP participants; however, random effects were the primary focus given the heterogeneity of our outcome and expected heterogeneity of the study populations and their exposures. Effects were estimated for FPG, PPG, HbA1c, and HOMA-IR with 95% CIs between pre- and post-intervention. All analyses were conducted to present a negative MD as a favorable intervention (i.e., lowering of measures of dysglycemia). Heterogeneity was assessed using Tau^2^ and I^2^, as well as the calculation of prediction intervals (PI). Where heterogeneity was high or of interest due to population/study heterogeneity (I^2^ > 50%), subgroup analysis and meta-regression were performed (if ≥2 RCTs were included in the meta-analysis). Planned subgroup analysis included: maternal age, gestational age, maternal BMI, country of study, diabetes diagnostic criteria, and study duration). Forest plots were created using R Statistical Software (v2022.07.2+576; RStudio Team 2022).

### 2.4. Grading the Evidence

The Grading of Recommendations Assessment, Development and Evaluation (GRADE) tool was used to improve the interpretability of results data, evaluate the certainty of the evidence, and determine the strength of the review conclusions [25]. Evidence of an effect can be graded either ‘very low’, ‘low’, ‘moderate’, or ‘high’ based on evaluation outcomes in five domains—overall risk of bias, inconsistency, indirectness, imprecision, and other considerations.

## 3. Results

**Figure 1:**
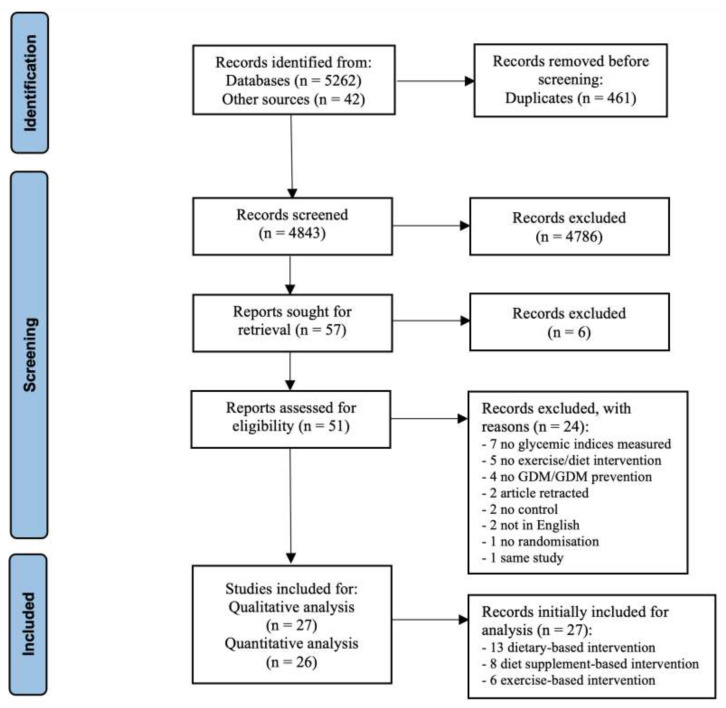
PRISMA flow diagram of study selection adapted from Page MJ, et al. (2020) [22].

A total of 5304 studies were identified through database searches and other sources. After de-duplication, 4843 were assessed for a title- and abstract screening. Of these, 51 reports progressed to full-text screening, of which 24 were excluded for not meeting the inclusion criteria (Figure 1). In total, 24 RCTs and 3 randomized crossover trials were included in the systematic review, and 23 RCTs and 3 randomized crossover trials in the meta-analysis, comprising a total of 1653 individuals with gestational diabetes. No studies including other types of diabetes during pregnancy, i.e., pre-existing T1D or T2D, were identified. The RCTs were classified according to the intervention type of the study as a nutritional supplement- (n  =  8, Table 1), dietary- (n  =  13, Table 2), or exercise-based (n = 6, Table 3). A nutritional supplement is defined as a product intended for ingestion that contains a “dietary ingredient”, which is a concentrated source of a vitamin or mineral, or other substance with a nutritional or physiological effect, alone or in combination, intended to supplement the diet and is sold in dose form. Of the studies retained for analysis, nutritional supplement interventions focused on alpha-lipoic acid, probiotic, ginger, fish oil, or a combination of zinc and vitamin intake versus a placebo. Dietary interventions primarily focused on higher complex CHO/lower GI, restricted energy intake, and Dietary Approaches to Stop Hypertension (DASH) diets versus a standard care diet. Finally, exercise interventions focused on brisk walks, resistance exercise, home-based exercise, and moderate-intensity aerobics versus standard antenatal care.

### 3.1. Nutritional Supplement-Based Interventions

**Figure 2:**
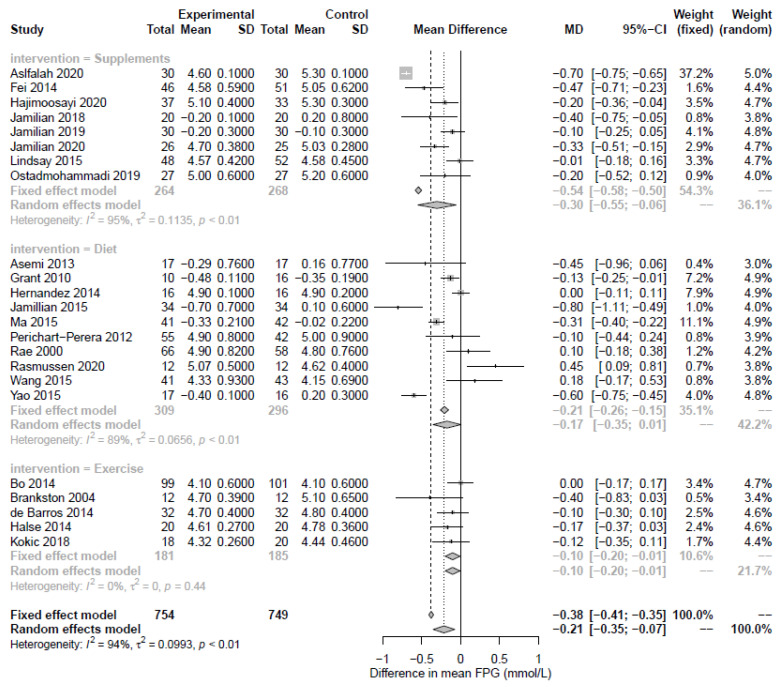
Forest plot of fasting plasma glucose (mmol/L). Fixed and random-effect meta-analysis of included studies. Overall test for effect of any lifestyle intervention (with all studies; n = 23) and subgroup analysis by intervention type—nutritional supplements (n = 8), diet (n = 10), and exercise (n = 5)—are presented. SD, standard deviation; CI, confidence interval.

**Figure 3:**
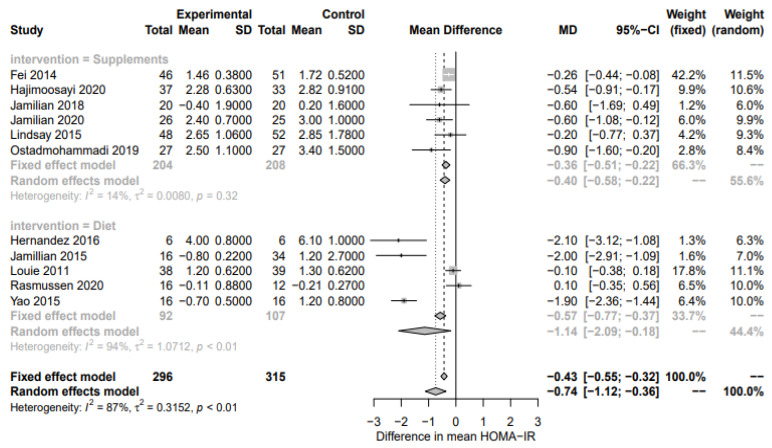
Forest plot of HOMA-IR. Fixed and random-effect meta-analysis of included studies. Overall test for effect of any lifestyle intervention (with all studies; n = 23) and subgroup analysis by intervention type—nutritional supplements (n = 8), diet (n = 10), and exercise (n = 5)—are presented. SD, standard deviation; CI, confidence interval.

In total, 8 RCTs were identified that reported on the effect of nutritional supplements on markers of dysglycemia in a total of 541 participants. Of these, 8 reported fasting glucose, 1 reported PPG, 1 reported HbA1c, and 6 reported HOMA-IR. The supplement interventions focused on alpha-lipoic acid, probiotic, ginger, fish oil, or combination of zinc and vitamin intake versus a placebo. Supplement-based interventions significantly reduced FPG (8 RCTs, −0.30 mmol/L; 95% CI −0.55, −0.06; *p* = 0.02; I2  =  95%, Figure 2), with high heterogeneity. Only 1 RCT reported PPGR and HbA1c, so no meta-analysis was performed. HOMA-IR was significantly reduced by supplement-based interventions (6 RCTs, −0.40; 95% CI −0.58, −0.22; *p* < 0.0001; I2  =  14%, Figure 3). The funnel plots for FPG and HOMA-IR did not indicate asymmetry (Figures S1 and S2).

Subgroup analysis of nutritional supplement-based interventions—including maternal age, gestational age, body weight, GDM diagnostic criteria, and geographic region—for FPG did not demonstrate changes in effecting size greatly from the overall analysis (Table 4), but it did suggest that studies initiated later in pregnancy and in non-Western countries may be less effective. For HOMA-IR, our analysis suggests supplement-based interventions initiated earlier in pregnancy, in younger women, and in non-Western countries are most likely to be effective. With only 1 RCT of the nutritional supplement intervention studies reporting HbA_1c_ and PPG, subgroup analyses for these outcomes were not performed.

### 3.2. Diet-Based Interventions

**Figure 4:**
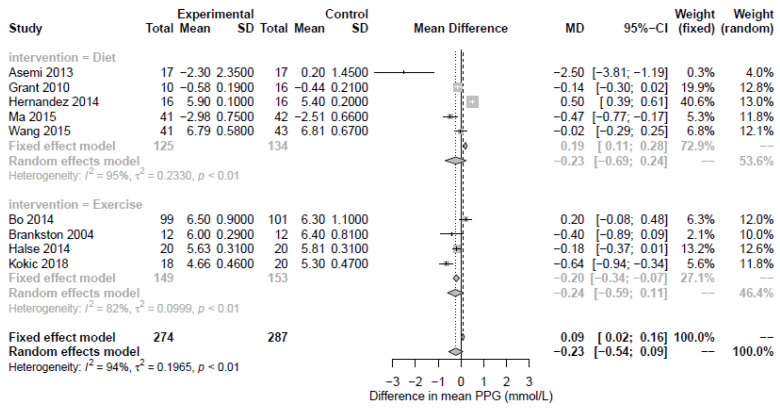
Forest plot of postprandial plasma glucose (mmol/L). Fixed and random-effect meta-analysis of included studies. Overall test for effect of any lifestyle intervention (with all studies; n = 23) and subgroup analysis by intervention type—nutritional supplements (n = 8), diet (n = 10), and exercise (n = 5)—are presented. SD, standard deviation; CI, confidence interval.

**Figure 5:**
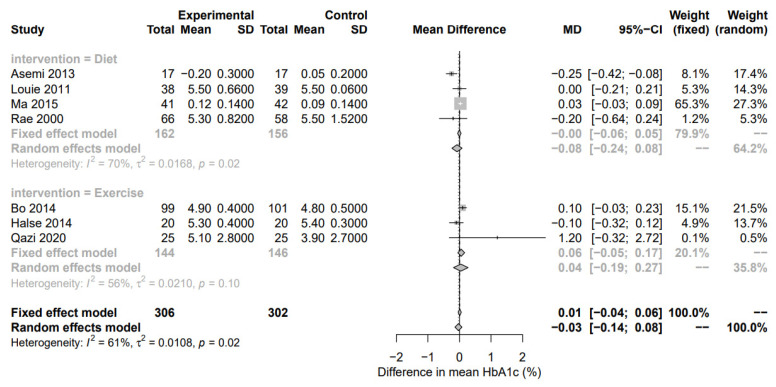
Forest plot of glycated hemoglobin (%). Fixed and random-effect meta-analysis of included studies. Overall test for effect of any lifestyle intervention (with all studies; n = 23) and subgroup analysis by intervention type—nutritional supplements (n = 8), diet (n = 10), and exercise (n = 5)—are presented. SD, standard deviation; CI, confidence interval.

In total, 10 RCTs and 2 crossover trials were reported on the effect of diet on markers of dysglycemia (n = 676 participants). 10 studies reported fasting glucose, 5 reported PPG, 4 reported HbA1c, and 5 reported HOMA-IR. The dietary interventions primarily focused on higher complex CHO/lower GI, restricted energy intake, and DASH versus a standard care diet. HOMA-IR was significantly reduced by diet interventions (HOMA-IR; n = 5 RCTs, MD −1.15; 95% CI −2.36, −1.44; *p* = 0.02; I2 = 94%, Figure 3) while fasting plasma glucose, although not significant, suggested some evidence of an effect, albeit with high heterogeneity (n = 10 RCTs, MD −0.17; 95% CI −0.35, 0.01; *p* = 0.06; I2 = 89%, Figure 2). The shape of the funnel plots for FPG and HOMA-IR did not suggest symmetry (Figures S3 and S4). Postprandial glucose and HbA1c were not significantly associated with diet-based interventions (n = 5 RCTs, MD −0.23; 95% CI −0.69, 0.32; *p* = 0.34; I2 = 95% and n = 4 RCTs, MD −0.08; 95% CI −0.23, 0.08; *p* = 0.34; I2 = 70%, respectively, Figure 4 and Figure 5).

Subgroup analysis for FPG and PPG did not differ greatly from the main overall analysis (Table 5). However, for HbA1c, subgroup analysis suggested that the effectiveness of diet interventions is primarily driven by its effect in overweight individuals when the ADA criteria are not used (2 RCTs; −0.24%; 95% CI −0.40, −0.08; *p* = 0.003; I2 = 0%, Table 5). Additionally, subgroup analysis of diet on HOMA-IR suggested that diet is most effective in longer studies with younger participants at an earlier gestational age, and in non-Western countries that do not use the ADA criteria.

### 3.3. Exercise-Based Interventions

In total, 5 RCTs and 1 crossover trial reported on the effect of exercise on markers of dysglycemia (n = 416 participants). Of these, 5 reported fasting glucose, 4 reported PPG, 1 reported HbA1c, and none reported HOMA-IR. The exercise interventions focused on brisk walks, resistance exercise, home-based exercises, and moderate-intensity aerobics versus standard antenatal care. Fasting glucose was significantly reduced by exercise-based interventions (n = 5 RCTs, MD −0.10; 0% CI −0.20, −0.01; *p* = 0.04; I2 = 0%, Figure 2). However, postprandial glucose and HbA1c were not significantly affected by exercise-based interventions (n = 4 RCTs, ES −0.17; 95% CI −0.35, 0.01; *p* = 0.17; I2 = 82% and n = 3 RCTs, ES 0.04; 95% CI −0.19, 0.27; *p* = 0.73; I2 = 56%, respectively, Figure 4 and Figure 5). Only 1 RCT reported HOMA-IR, therefore no meta-analysis was performed. The funnel plot for FPG did not indicate asymmetry (Figure S5).

Subgroup analysis of exercise-based interventions by moderators of gestational dysglycemia—maternal age, gestational age, and body weight—suggested that maternal age, gestational age, and pre-pregnancy weight may modify the effectiveness of exercise-based interventions but not significantly (Table 6). For PPG and HbA1c, subgroup analysis did not change effect sizes or heterogeneity (Table 6).

### 3.4. Risk of Bias Assessment

Risk of bias assessment across the studies indicated low risk/some concerns for the majority of RCTs (12 studies and 14 studies, respectively) due to a lack of information on randomization concealment and blinding of outcome assessors (Supplemental Table S2). There was one study that was considered ‘high risk’ due to concerns in three or more domains—i.e., lack of information on randomization concealment, blinding of outcome assessors, and *p*-values/standard deviations. The study that fell into the ‘high risk’ category, Valentini et al. (2012), was removed for these reasons and the lack of data on *p*-values from the meta-analysis.

### 3.5. Grading the Evidence

The GRADE assessments for all analyses are summarized in Supplemental Tables S3–S5. The assessment for dietary-based interventions revealed a ‘moderate’ grade for HOMA-IR, and ‘low’ and ‘very low’ grades for fasting glucose, PPG, and HbA1c in GDM, which were most commonly downgraded due to inconsistency and imprecision of these outcomes. Evidence on nutritional supplement-based interventions was graded as ‘moderate’ for HbA1c and HOMA-IR, and ‘low’ and ‘very low’ for fasting glucose and PPG, mainly due to low ratings for consistency, directness, and precision. Furthermore, assessment for exercise-based interventions revealed a ‘moderate’ grade for fasting glucose, and ‘very low’ and ‘low’ grades for PPG, HbA1c, and HOMA-IR in GDM, due to inconsistency, indirectness, and imprecision of these outcomes.

## 4. Discussion

To the best of our knowledge, this is the first systematic review and meta-analysis with a comprehensive analysis of the impact of these three types of lifestyle intervention in GDM on maternal glucose. A total of 24 RCTs and 3 randomized crossover trials were identified to investigate the magnitude and generalizability of the effects of lifestyle on glycemic control in women with GDM. Of the 5304 records identified, only studies in women that developed GDM were identified, and no RCTs or crossover trials in pregnant women with pre-existing T1D or T2D were identified that reported on maternal glucose. The studies in women with GDM reported on the effects of diet (whole foods, n = 13), nutritional supplements (n = 8), or exercise-based (n = 6) interventions. Compared with previous systematic reviews in women with GDM published before 2019, this review included 5 more RCTs and conducted several subgroups to control for heterogeneity, including maternal age, BMI, ethnicity, duration of intervention, intervention types, and diagnosis guidelines used. These subgroups were defined to better characterize and present the effects of lifestyle modifications in diverse populations. Our results suggest that supplement-based interventions improved both FPG and HOMA-IR, while diet- and exercise-based interventions only improved one glycemic measure (HOMA-IR or FPG, respectively).

### 4.1. Nutritional Supplement-Based Interventions

In total, 8 RCTs (n = 541 participants) reported on the effects of nutritional supplements on markers of dysglycemia. Supplement interventions focused on alpha-lipoic acid, probiotic, ginger, fish oil, or zinc and vitamin supplements versus placebo. Overall, supplement-based interventions significantly improved FPG and HOMA-IR in numerous studies, and subgroup analysis suggested that common moderators of GDM risk do not modify the effectiveness of nutritional supplements on dysglycemia, except for maternal age and normal body weight, which could be important when considering nutritional supplement interventions. Therefore, maternal age and normal body weight could be considered as moderators. Unfortunately, the effect of supplement-based interventions on PPG and HbA1c was reported in only 1 RCT and could not be generalized.

Meta-analysis of RCTs on the effects of probiotics on glycemia in pregnancy by Pan et al. (2021) indicated that probiotic supplements improved FPG level (14 RCTs) and insulin resistance (HOMA-IR, 13 RCTs), specifically in GDM and healthy pregnant women, which is in trend with our results regarding nutritional supplements and improved levels of FPG and HOMA-IR [52]. Maternal age is a known confounder of glucose status with dysglycemic individuals typically older [53]. Our results suggest that nutritional supplements are less effective in reducing insulin resistance in the higher maternal age subgroup, as this group might have more severe dysglycemia. The exact mechanisms of probiotics on glycemic control remain unknown. Another meta-analysis (5 RCTs) by Ojo et al. (2019) concluded that vitamin D supplementation decreased FPG [54]. A review by Qu et al. (2022) on magnesium supplementation found significant improvement in glucose metabolism and insulin sensitivity (FPG, insulin) in addition to the specific marker of oxidative stress TAC [55]. While the mechanisms of vitamin D and magnesium on dysglycemia are not certain, potential mechanisms could include: (1) direct action on ß-cell function; (2) regulation of intracellular calcium and glucose transport, and (3) reduction of systemic inflammation associated with insulin resistance [55,56].

Our results confirm that nutritional supplements can reduce fasting glucose and insulin resistance, which underlines the difficulty of generalizability due to the heterogeneity and variety of nutritional supplements and the limited evidence regarding their effect on post-prandial and long-term estimates of dysglycemia (i.e., PPG and HbA1c). Based on the findings, future studies with a more uniform nutritional supplementation approach are warranted to make an informed recommendation for care guidelines on which supplements should be included and for how long for diabetes management.

### 4.2. Diet-Based Interventions

In total, 10 RCTs and two randomized crossover trials reported on the effect of diet on markers of dysglycemia (n = 676 participants). The dietary interventions primarily focused on higher complex CHO/lower GI, restricted energy intake, and Dietary Approaches to Stop Hypertension (DASH) diets versus a standard care diet. The trial by Valentini et al. (2012) was excluded from the meta-analysis due to serious bias concerns. Our analysis concluded that dietary interventions are advantageous for controlling HOMA-IR during pregnancy in women with GDM, with potential improvements in FPG as well. Subgroup analysis suggested that common moderators of GDM risk do not modify the effectiveness of dietary interventions on dysglycemia, except for lower maternal age, ADA diagnostic criteria, and a non-western country. Pregnant women with lower maternal age are less likely to suffer from severe dysglycemia; thus, interventions might be more effective and insulin resistance might be easier to improve in this subgroup [53]. All non-western country studies used ADA guidelines as diagnostic criteria, suggesting a disagreement of diagnostic criteria as a previous study found IADPSG (i.e., ADA) criteria more favorable than NICE for identification of adverse pregnancy outcomes among Asian and Hispanic women, while they are comparable to NICE among White women [57]. Furthermore, studies with lower glucose thresholds for GDM selection may have less impact.

Prescribing a low-, reduced-carbohydrate diet for pregnant women with GDM as a first-line treatment has been linked to reduced FPG, decreased risk of postprandial hyperglycemia, and reduced risk of requiring insulin to manage dysglycemia [9,58,59]. The previous review on a variety of modified dietary interventions and maternal glycemia by Yamamoto et al. (2018) pooled results from 18 RCTs, including women with GDM, impaired glucose tolerance, or hyperglycemia. Their meta-analysis found a moderate effect of dietary interventions on maternal glycemic outcomes, including changes in FPG (13 RCTs), PPG (9 RCTs), and need for medication treatment, and a nearly significant effect on HOMA-IR (4 RCTs) [6]. We found a potential advantageous effect of dietary interventions on FPG (10 RCTs) but were unable to find an effect on PPG (5 RCTs); this is possibly due to our SRMA only including studies published after 2000 where actual diets were prescribed to the participants; thus, fewer studies were available. Furthermore, our meta-analysis, including 1 more RCT (4 vs. 5 RCTs), did demonstrate a significant effect on HOMA-IR. Both Yamamoto et al., (2018) and our analysis demonstrated a high heterogeneity, which could be explained by differences in baseline FPG or PPG levels having influenced the glucose-related outcomes. These improvements in glycemic markers could be the result of dietary intervention’s ability to reduce spikes in postprandial glucose responses [60]. Our meta-analysis supports current recommendations that prescribe dietary interventions to manage dysglycemia during pregnancy. Future work that accounts for dietary adherence may allow for better clarity of the effectiveness and feasibility of distinct diets.

### 4.3. Exercise-Based Interventions

In addition to dietary modifications, exercise is a vital component in GDM management. The ADA and NICE guidelines recommend that pregnant women with GDM, who have no medical contraindications, should undertake brisk walks for 20 min/day or moderate exercise consisting of 30 min most days of the week as part of GDM treatment [9,10]. In total, our meta-analysis included 5 RCTs and 1 randomized crossover trial that reported on the effects of exercise on markers of dysglycemia in a total of 416 participants. The exercise interventions focused on brisk walks, resistance exercise, home-based exercise, and moderate-intensity aerobics exercise versus standard antenatal care. Our pooled analysis demonstrated that exercise interventions are advantageous for controlling FPG during pregnancy in women with GDM. Subgroup analysis for this type of intervention was limited due to fewer included studies, and studies included could not be divided into subgroups for some of the categories. Lower maternal age, later gestational age, and normal weight could be considered as moderators. Previous published systematic reviews and meta-analyses by Brown et al. (2017) (11 RCTs) and Cremona et al. (2018) (12 RCTs) on aerobic/resistance exercise or combination for women with GDM reported that exercise interventions were associated with reduced FPG and PPG concentrations compared with conventional interventions [61,62]. Another systematic review by Allehdan et al. (2019) (8 RCTs) showed evidence that dietary management plus aerobic or resistance exercise interventions improved glycemic outcomes and lowered FPG and PPG levels for women with GDM compared with dietary management alone [3]. Both aerobic and resistance exercise are beneficial for improving glycemic control, and it is optimal to do both types of exercise [63]. Previous research has established that exercise increases the rate of glucose uptake into the skeletal muscle, this occurs during exercise and for some hours post-exercise. The increased uptake is a result of the translocation of glucose transport protein, thereby increasing the sites where glucose can diffuse into the muscle cells [63,64]. Exercise also stimulates glucose uptake by promoting insulin action via increasing the use of intracellular fatty acids and improving insulin sensitivity, and stimulating glucose uptake independently from insulin sensitivity [65]. These confirmed effects and associations of exercise with improved insulin sensitivity may explain the improvement in FPG levels shown in our results.

This meta-analysis shows an advantageous effect of exercise on FPG, which is in agreement with previously conducted studies but did not report a significant effect on PPG or HbA1c. As such, future studies are needed to determine the effect of exercise interventions on PPG, HbA1c, and HOMA-IR. Overall, larger-effect sizes, higher-graded evidence, and less heterogeneity were reported in the supplement-based interventions compared to diet- and exercise-based interventions. This is likely due to the ease of adherence and standardization of supplements compared to diet and exercise, which are likely more susceptible to changes in routine and circumstance (e.g., extended work hours, family commitments, sickness, etc.). As such, diet- and exercise-based interventions may require greater personalization and prescribed flexibility to suit patient needs.

### 4.4. Strengths and Limitations

Six of the included studies were pilot studies or underpowered to determine significant differences for the primary outcomes of this review [35,37,43,45,47,51]. Furthermore, subgroup analysis based on common moderators of GDM risk could not be performed for some of the outcomes. Due to different intervention strategies within each of the lifestyle categories, it was not possible to perform a network analysis. Moreover, the short duration of some of the interventions and the late gestational age, at which the interventions were started, may have limited their impact on glycemic outcomes. Finally, a very- or low-GRADE quality score for most outcomes (supplements: FPG and PPG; diet: FPG, PPG, and HbA1c; exercise: PPG, HbA1c, and HOMA-IR) due to limitations in the design of included studies (e.g., allocation concealment, lack of blinding of either outcome assessors or participants, reporting of adherence to the intervention) could explain the lack of difference between intervention and control. The strengths of this review should be noted, as far as we know, this is the first SRMA that shows the benefits of supplement-, dietary-, and exercise-based interventions on measures of glycemic control in GDM, including more recent studies not included by the preceding SRMAs [26,28,31,42,51]. Overall this SRMA included a large number of participants with varied backgrounds and examines the effectiveness of lifestyle interventions on maternal glycemic control, ultimately reducing the risk of adverse perinatal outcomes.

## 5. Conclusions

This meta-analysis highlights the key role of nutritional supplements, diet, and exercise in the management of GDM and shows promising advantageous effects on measures of glycemia—i.e., FPG, PPG, and HOMA-IR. HOMA-IR had the largest significant effect sizes, least heterogeneity, and best GRADE. Future RCTs should consider incorporating HOMA-IR as an outcome in the study design and perhaps should combine the different intervention types. Furthermore, no RCTs in women with pre-existing T1D or T2D in pregnancy were identified. There is a prominent need for large, well-designed RCTs that clarify the most effective lifestyle intervention or a combination across a range of outcomes in women with all diabetes types during pregnancy and ideally incorporate longer-term outcomes in mothers and offspring, to eventually develop more suitable lifestyle recommendations for women with DIP.

## Figures and Tables

**Table 1:** Summary of RCTs investigating effect of nutritional supplement-based interventions on glycemic indices in GDM.

	Country	n	Estimated Sample Size	Definition of GDM (Diagnostics Criteria)	Intervention Duration	Design Intervention Description	Participant Characteristics	Outcomes Measures
*Aslfalah et al., (2020)* [26]	Iran	60 (n = 30 for both groups)	Not reported	American Diabetes Association guidelines	8 weeks	RCT double-blinded *Intervention*: received ALA (100 mg/day) *Control*: received cellulose acetate (100 mg/day)	***Age*** *Intervention*: 30.96 ± 0.93 *Control*: 31.10 ± 0.92 ***Wks of gestation at baseline*** *Intervention*: 26.28 ± 0.23 *Control*: 26.51 ± 0.24 ***BMI (pre-pregnancy)** Intervention*: 26.64 ± 0.71 *Control*: 26.95 ± 0.73	Fasting plasma glucose and glycated haemoglobin
*Fei et al., (2014)* [27]	China	97 (n = 46 for I and n = 51 for C)	Not reported	National Diabetes Data group guidelines	8 weeks	RCT *Intervention*: treated with the combination of insulin, regular diet, and soybean oligosaccharides (SBOS) *Control*: regular diet and insulin treatment	Not reported	Fasting plasma glucose and HOMA index
*Hajimoosayi et al., (2020)* [28]	Iran	70 (n = 37 for I and n = 33 for C)	Considering a 99% CI, power of 90%, and 30% dropout rate, a sample size of 38 per group was determined.	International Association of the Diabetes in Pregnancy Study Group guidelines	6 weeks	RCT double-blinded *Intervention*: received 126 tablets of ginger, *Control*: received 126 tablets of placebo	***Age*** *Intervention*: 29.68 ± 5.05 *Control*: 31.15 ± 5.26 ***Wks of gestation at baseline*** *Intervention*: 27.72 ± 3.6 *Control*: 27.78 ± 3.60 ***BMI (at baseline)*** *Intervention*: 29.60 ± 3.6 *Control*: 29.50 ± 4.30	Fasting plasma glucose, postprandial glucose and HOMA index
*Jamilian et al., (2018)* [29]	Iran	40 (n = 20 for both groups)	Not reported	American Diabetes Association guidelines	6 weeks	RCT double-blind *Intervention*: 1000 mg fish oil capsules, containing 180 mg eicosapentaenoic acid and 120 mg docosahexaenoic acid twice a day *Control*: placebo	***Age*** 30.8 ± 2.4 ***Wks of gestation at baseline*** 25.3 ± 1.1 ***BMI (at baseline)*** 27.0 ± 3.1	Fasting plasma glucose and HOMA index
*Jamilian et al., (2019)* [30]	Iran	60 (n = 30 for both groups)	Considering a type 1 error of 5%, power of 80%, and hs-CRP mean distinction of 3.2 mg/L as outcome, a sample size of 25 per group was determined.	American Diabetes Association guidelines	6 weeks	RCT double-blind *Intervention*: magnesium-zinc-calcium-vitamin D supplements *Control*: placebo	***Age*** *Intervention*: 27.7 ± 4.0 *Control*: 29.1 ± 4.1 ***BMI (at baseline)*** *Intervention*: 25.8 ± 3.7 *Control*: 25.3 ± 2.5	Fasting plasma glucose
*Jamilian et al., (2020)* [31]	Iran	60 (n = 26 for I and n = 25 for C)	Considering a type 1 error of 5%, power of 80%, and PPAR-y change of 0.20 as outcome, a sample size of 25 per group was determined.	American Diabetes Association guidelines	6 weeks	RCT double-blinded *Intervention*: 2 × 1000 mg/d n-3 fatty acids from flaxseed oil containing 400 mg α-linolenic acid in each capsule *Control*: placebo	***Age*** *Intervention*: 29.5 ± 5 *Control*: 28.5 ± 4.1 ***BMI (at baseline)** Intervention*: 28.9 ± 4.8 *Control*: 27.3 ± 4.1	Fasting plasma glucose and HOMA index
*Lindsay et al., (2015)* [32]	Ireland	100 (n = 48 for I and n = 52 for C)	Considering a type 1 error of 5%, power of 80%, and 0.4 mmol/L reduction in fasting plasma glucose as outcome, a sample size of 50 per group was determined.	Based on a 100 g-oral glucose tolerance test (Carpenter and Coustan, 1982)	Diagnosis until delivery	RCT double-blinded *Intervention*: daily probiotic (Lactobacillus salivarius UCC118) from diagnosis until delivery *Control*: placebo capsule from diagnosis until delivery	***Age*** *Intervention*: 33.5 ± 5.0 *Control*: 32.6 ± 4.5 ***Wks of gestation at baseline*** *Intervention*: 29.8 ± 2.5 *Control*: 29.5 ± 2.4 ***BMI (at baseline)*** *Intervention*: 29.06 ± 6.70 *Control*: 28.94 ± 5.79	Fasting plasma glucose and HOMA index
*Ostadmohammadi et al., (2019)* [33]	Iran	54 (n = 27 for both groups)	Not reported	American Diabetes Association guidelines	6 weeks	RCT double-blind *Intervention*: 233 mg/day Zinc Gluconate plus 400-IU/day vitamin E supplements *Control*: placebo	***Age*** *Intervention*: 31.1 ± 5.1 *Control*: 30.5 ± 3.1 ***Wks of gestation at baseline*** *Intervention*: 25.7 ± 1.40 *Control*: 25.3 ± 1.3 ***BMI (at baseline)** Intervention*: 29.3 *Control*: 28.5	Fasting plasma glucose, postprandial glucose and HOMA index

**Table 2:** Summary of RCTs and crossover studies investigating effect of diet-based interventions on glycemic indices in GDM.

Author, Year (Ref.)	Country	n	Estimated Sample Size	Definition of GDM (Diagnostics Criteria)	Intervention Duration	Design Intervention Description	Participant Characteristics	Outcomes Measures
*Asemi et al., (2013)* [34]	Iran	34 (n = 17 for both groups)	Considering a type I error of 5%, power of 80% and serum HDL cholesterol levels as outcome, a sample size of 16 per group was determined.	American Diabetes Association guidelines	4 Weeks	RCT *Intervention*: DASH diet *Control*: control diet contained 45–55% carbohydrates, 15–20% protein and 25–30% total fat	**Age** *Intervention*: 30.7 ± 6.7 *Control*: 29.4 ± 6·2 **BMI (at baseline)** *Intervention*: 29.0 ± 3.2 *Control*: 31.4 ± 5.7	Fasting plasma glucose, postprandial glucose and glycated haemoglobin
*Grant et al., (2011)* [35]	Canada	26 (n = 10 for I and n = 16 for C for GDM) (IGTP; n = 12)	Considering 85% power and to detect a difference of 0.6 mmol/L in capillary glucose between groups, a sample size of 50 was determined.	Canadian Diabetes Association guidelines	~8 weeks	RCT *Intervention*: low glycemic index dietary intervention as a supplement to the standard medical nutrition therapy (Canadian guidelines) *Control*: standard medical nutrition therapy (Canadian guidelines)	**Age** *Intervention*: 34 ± 0.1 *Control*: 34 ± 1.1 **Wks of gestation at baseline** *Intervention*: 29 ± 0.7 *Control*: 29 ± 0.5 **BMI (pre-pregnancy)** *Intervention*: 27 ± 1 *Control*: 26 ± 1	Fasting plasma glucose, Postprandial glucose and glycated haemoglobin
*Hernandez et al., (2014)* [36]	USA	16	Considering a type 1 error of 5%, power of 80%, and AUC as outcome, a sample size of 16 was determined.	American College of Obstetricians and Gynaecologists guidelines	3 days	Randomized crossover *Intervention*: Higher complex CHO/Lower fat diet *Control*: conventional low-carbohydrate/higher-fat diet	**Age** 28.4 ± 1.0 **Wks of gestation at baseline** 31.2 ± 0.5 **BMI (pre-pregnancy)** 30.6 ± 1.3	Fasting plasma glucose
*Hernandez et al., (2016)* [37]	USA	12 (n = 6 for both groups)	Not reported	Based on a 100 g-oral glucose tolerance test (Carpenter and Coustan, 1982)	~7 weeks	RCT *Intervention*: a higher–complex carbohydrate/lower-fat diet (60% carbohydrate/25% fat/15% protein) *Control*: conventional low-carbohydrate/higher-fat diet (40% carbohydrate/45% fat/15% protein)	**Age** *Intervention*: 30 ± 1.0 *Control*: 28 ± 2.0 **Wks of gestation at enrolment** *Intervention*: 31.7 ± 1.0 *Control*: 31.2 ± 0.4 **BMI (at baseline)** *Intervention*: 34.3 ± 1.6 *Control*: 33.4 ± 1.4	HOMA index
*Jamilian et al., (2015)* [38]	Iran	68 (n = 34 for both groups)	Considering the type 1 error of 5% power of 80%, a sample size of 28 per group was determined.	American Diabetes Association guidelines	6 weeks	RCT *Intervention*: soy diet containing the same amount of protein with 35% animal protein, 35% soy protein, and 30% other plant proteins *Control*: control diet containing 0.8-g/kg protein (70% animal and 30% plant proteins)	**Age** *Intervention*: 28.2 ± 4.6 *Control*: 29.3 ± 4.2 **Wks of gestation at baseline** *Intervention*: 29 ± 0.7 *Control*: 29 ± 0.5 **BMI (at baseline)** *Intervention*: 28.9 ± 5.0 *Control*: 28.4 ± 3.4	Fasting plasma glucose and HOMA index
*Louie et al., (2011)* [39]	Australia	77 (n = 38 for I and n = 39 for C)	Considering power of 80% and to detect a ∼260 g difference in birth weight, a sample size of 60 per group was determined.	Australasian Diabetes in Pregnancy Society (ADIPS) guidelines	~6–7 weeks	RCT Both diets consisted of similar protein (15–25%), fat (25–30%), and carbohydrate (40–45%) content *Intervention*: an Low- glycemic index (target GI ≤ 50) *Control*: a high-fibre content and moderate GI, similar to the Australian population average (target GI ∼60)	**Age** *Intervention*: 34.0 ± 4.1 *Control*: 32.4 ± 4.5 **Wks of gestation at baseline** *Intervention*: 29.0 ± 4.0 *Control*: 29.7 ± 3.5 **BMI (pre-pregnancy)** *Intervention*: 23.9 ± 4.4 *Control*: 24.1 ± 5.7	HOMA index and glycated haemoglobin
*Ma et al., (2014)* [40]	China	83 (n = 41 for I and n = 42 for C)	Not reported	Chinese Medical Association and the American Diabetes Association guidelines	Every 2 weeks from 24–26 weeks of gestation to delivery	RCT *Intervention*: intensive low-GL intervention *Control*: individualized general dietary intervention	**Age** *Intervention*: 30.1 ± 3.8 *Control*: 30.0 ± 3.5 **Wks of gestation at baseline** *Intervention*: 27.5 ± 1.1 *Control*: 27.9 ± 1.1 **BMI (pre-pregnancy)** *Intervention*: 21.90 ± 3.14 *Control*: 21.15 ± 2.75	Fasting plasma glucose, postprandial glucose, and glycated haemoglobin
*Perichart-Perera et al., (2012)* [11]	Mexico	107 (n = 55 for I and n = 42 for C)	Considering the type 1 error of 5% power of 80%, and 10 mg/dL difference in glucose, a sample size of 32 per group was determined.	American Diabetes Association guidelines	Not reported	RCT *Intervention*: Women received an individual food plan based on CHO restriction (only low glycemic index (GI) carbohydrates (CHO)) *Control*: Women received an individual food plan based on CHO restriction (all types of CHO)	**Age** *Intervention*: 32.3 ± 4.8 *Control*: 31.8 ± 5.3 **Wks of gestation at enrolment** *Intervention*: 22.5 ± 4.9 *Control*: 20.7 ± 6.7 **BMI at baseline** *Intervention*: 30.5 ± 5.2 *Control*: 32.0 ± 6.3	Fasting plasma glucose
*Rae et al., (2000)* [41]	Australia	124 (n = 66 for I and n = 58 for C)	Considering the type 1 error of 5% power of 80%, and frequency of insulin and macrosomia use as outcomes, a sample size of 60 per group was determined.	Not reported	Treatment until delivery (not further specified)	RCT *Intervention*: a moderately energy restricted diabetic diet providing between 1590–1776 kilocalories. Representing 70% of the RDI for pregnant women (National Health and Medical Research Council of Australia) *Control*: a diabetic diet which was not energy restricted	***Age*** *Intervention*: 30.2 *Control*: 30.8 ***Wks of gestation at diagnosis*** *Intervention*: 28.1 ± 5.8 *Control*: 28.3 ± 4.6 ***BMI (at diagnosis)*** *Intervention*: 37.9 ± 0.7 *Control*: 38.0 ± 0.7	Fasting glucose and glycated haemoglobin
*Rasmussen et al., (2020)* [42]	Denmark	12	Considering the power of 80%, and to detect 5% between groups based on Dalfra (2013), a sample size of 12 was determined.	WHO diagnostic criteria	4 days	Randomised crossover Study Low carbohydrate morning intake vs. high carbohydrate morning intake	***Age*** 33.6 ***Gestational age*** 33.5 ***BMI (pre-pregnancy)*** 25.2	Fasting blood glucose
*Valentini et al., (2012)* [43]	Italy	20 (n = 10 for both groups)	Pilot study	American Diabetes Association guidelines	Not reported	RCT *Intervention*: an ethnic meal plan (EMP), a food plan that included dishes typical of the foreign women’s original countries *Control*: a standard meal plan (SMP) prepared according to the ADA guidelines	***Age*** *Intervention*: 28.9 ± 3.3 *Control*: 30.2 ± 4.7 ***BMI (pre-pregnancy) Intervention***: 25.7 ± 3.6 *Control*: 24.1 ± 4.7	Fasting plasma glucose, postprandial glucose and glycated haemoglobin
*Wang et al., (2015)* [44]	China	84 (n = 41 for I and n = 43 for C)	Not reported	Based on a 75 g-oral glucose tolerance test	~6–8 weeks	RCT *Intervention*: an oil-rich diet, with sunflower oil (45–50 g daily) used as cooking oil *Control*: a low-oil diet, with sunflower oil (20 g daily) used as cooking oil	***Age*** *Intervention*: 30.29 ± 4.17 *Control*: 29.72 ± 4.64 ***Wks of gestation at baseline*** *Intervention*: 27.41 ± 1.52 *Control*: 27.34 ± 1.96 ***BMI (pre-pregnancy)** Intervention*: 21.36 ± 3.0 *Control*: 22.18 ± 3.60	Fasting plasma glucose and postprandial glucose
*Yao et al., (2015)* [45]	China	33 (n = 17 for I and n = 16 for C)	Considering a 75 g birthweight difference between groups, a sample size of 21 per group was determined.	American Diabetes Association guidelines	4 weeks	RCT *Intervention*: DASH diet *Control*: control diet including 45–55% carbohydrates, 15–20% protein and 25–30% total fat.	***Age*** *Intervention*: 30.7 ± 5.6 *Control*: 28.3 ± 5.1 ***Wks of gestation at baseline*** *Intervention*: 26.9 ± 1.4 *Control*: 25.7 ± 1.3 ***BMI (pre-pregnancy)** Intervention*: 29.6 ± 5.3 *Control*: 30.9 ± 4.3	Fasting blood glucose and HOMA index

**Table 3:** Summary of RCTs investigating effect of exercise-based interventions on glycemic indices in GDM.

Author, Year (Ref.)	Country	n	Estimated Sample Size	Definition of GDM (Diagnostics Criteria)	Intervention Duration	Design Intervention Description	Participant Characteristics	Outcomes Measures
*Bo et al., (2014)* [46]	Italy	200 (n = 99 for I and n = 101 for C)	Considering an effect size of 0.50, power of 95%, and a 10% reduction in fasting plasma glucose as outcome, a sample size of 200 was determined.	Based on a 75 g-oral glucose tolerance test	~12–14 weeks	2 × 2 design single-blinded All women were given the same diet (carbohydrates 48–50%, proteins 18–20%, fats 30–35%, fiber 20–25 g/day, no alcohol *Intervention*: received dietary recommendations *Control*: instructed to briskly walk 20-min/day	***Age*** *Intervention*: 35.9 ± 4.8 *Control*: 33.9 ± 5.3 ***BMI (pre-pregnancy)** Intervention*: 25.1 ± 4.6 *Control*: 24.8 ± 4.2	Fasting plasma glucose, postprandial glucose and HOMA index
*Brankston et al., (2004)* [47]	Canada	24 (n = 12 for both groups)	Considering a type 1 error of 5%, power of 80%, and insulin use reduced to 25% as outcome, a sample size of 32 per group was determined.	Canadian Diabetes Association guidelines	At least 4 weeks	RCT *Intervention*: circuit-type resistance training three times per week and same standard diet. *Control*: standard diabetic diet that consisted of 40% carbohydrate, 20% protein, and 40% fat.	***Age*** *Intervention*: 30.5 ± 4.4 *Control*: 31.3 ± 5.0 ***Wks of gestation at baseline*** *Intervention*: 29.0 ± 2.0 *Control*: 29.6 ± 2.1 ***BMI (pre-pregnancy)*** *Intervention*: 26.4 ± 7.1 *Control*: 25.2 ± 6.7	Fasting plasma glucose and postprandial plasma glucose
*de Barros et al., (2010)* [48]	Brasil	64 (n = 32 for both groups)	Considering a type 1 error of 5%, power of 80%, and insulin use required up to 20%, a sample size of 30 per group was determined.	Based on a 2 hr-75 g- or 3 hr-100 g- oral glucose tolerance test	~6 weeks	RCT *Intervention*: resistance exercise program *Control*: no resistance exercise program	***Age*** *Intervention*: 31.81 ± 4.87 *Control*: 32.40 ± 5.40 ***Wks of gestation at baseline*** *Intervention*: 31.56 ± 2.29 *Control*: 31.06 ± 2.30 ***BMI (pre-gestational)** Intervention*: 25.34 ± 4.16 *Control*: 25.39 ± 3.81	Fasting plasma glucose
*Halse et al., (2014)* [49]	Australia	40 (n = 20 for both groups)	Considering a type 1 error of 5%, power of 80%, and to detect a minimum 0.3 mM difference in fasting plasma glucose, a sample size of 20 per group was determined.	Based on a 75 g-oral glucose tolerance test (Australian criteria)	~6 weeks (until week 34 of pregnancy)	RCT *Intervention*: home-based exercise training in combination with conventional management *Control*: conventional management alone	***Age*** *Intervention*: 34 ± 5 *Control*: 32 ± 3 ***Wks of gestation at enrolment*** *Intervention*: 28.8 ± 0.8 *Control*: 28.8 ± 1 ***BMI (pre-pregnancy)** Intervention*: 26.4 ± 7.1 *Control*: 25.2 ± 6.7	Fasting plasma glucose, postprandial glucose and glycated haemoglobin
*Kokic et al., (2018)* [50]	Croatia	38 (n = 18 for I and n = 20 for C)	Not reported	International Association of the Diabetes and Pregnancy Study Groups guidelines	From the time of diagnosis of GDM until birth (minimum 6 weeks)	RCT single-blinded *Intervention*: standard antenatal care for GDM, and regular supervised exercise programme (two times per week 50–55 min; mixed exercises) plus daily brisk walks of at least 30 min. *Control*: only standard antenatal care for GDM.	***Age*** Intervention: 32.78 ± 3.83 Control: 31.95 ± 4.91 ***Wks of gestation at baseline*** *Intervention*: 22.44 ± 6.55 *Control*: 20.80 ± 6.05 ***BMI (at baseline)** Intervention*: 24.39 ± 4.89 *Control*: 25.29 ± 4.65	Fasting plasma glucose and postprandial glucose
*Qazi et al., (2020)* [51]	Pakistan	50 (n = 25 for both groups)	Considering a CI of 95% and power of 80%, a sample size of 27 per group was determined.	Based on a 75 g-oral glucose tolerance test	5 weeks	RCT *Intervention*: combination of moderate intensity aerobics, stabilization and pelvic floor muscles exercises twice a week for 5 weeks (40 min per session) along with dietary and medical interventions *Control*: only medical and dietary interventions with postural education	***Age*** *Intervention*: 34.36 ± 5.21 *Control*: 35.92 ± 5.24	Glycated haemoglobin

**Table 4:** Subgroup analysis of nutritional supplement vs. control interventions.

Category	Outcome Measure	RCTs (n)	MD	95% CI	*p*-Value	I^2^
Fasting Plasma Glucose (FPG, mmol/L)						
**Main analysis**	*Overall*	8	−0.30	(−0.55, −0.06)	**0.02**	95
**Maternal Age ^1^**	*<Mean age*	4	−0.33	(−0.76, 0.10)	0.13	96
	*≥Mean age*	3	−0.20	(−0.33, −0.07)	**0.002**	45
**Gestational Age ^2^**	*<28 weeks*	4	−0.39	(−0.72, −0.05)	**0.02**	93
	*≥28 weeks*	1	−0.01	(−0.18, 0.16)	0.905	NA
**Weight (pre-pregnancy) ^3^** **(kg/m^2^)**	*Normal weight (<25)*	5	−0.18	(−0.31, −0.05)	**0.005**	55
	*Overweight (≥25)*	1	−0.70	(−75, −0.65)	**<0.0001**	NA
**Diagnostic Criteria for GDM**	*ADA*	5	−0.35	(−0.66, −0.04)	**0.03**	94
	*Other*	3	−0.30	(−0.39, 0.02)	0.08	79
**Geographic Region**	*Western country*	1	−0.01	(−0.18, 0.16)	0.905	NA
	*Non-western country*	7	−0.35	(−0.59, −0.10)	0.005	94
HOMA-IR						
**Main analysis**	*Overall*	6	−0.40	(−0.58, −0.22)	**<0.0001**	14
**Maternal Age ^1^**	*<Mean age*	2	−0.56	(−0.86, −0.27)	**0.002**	0
	*≥Mean age*	3	−0.51	(−0.96, −0.05)	**0.03**	15
**Gestational Age ^2^**	*<28 weeks*	3	−0.62	(−0.93, −0.30)	**0.0001**	0
	*≥28 weeks*	1	−0.2	(−0.77, 0.37)	0.501	NA
**Diagnostic Criteria for GDM**	*ADA*	3	−0.68	(−1.05, −0.31)	**0.0003**	0
	*Other*	3	−0.30	(−0.46, −0.15)	**0.0001**	0
**Geographic Region**	*Western country*	1	−0.2	(−0.77, 0.37)	0.501	NA
	*Non-western country*	5	−0.45	(−0.67, −0.23)	**<0.0001**	27

^1^ Maternal age not reported in 5 studies. ^2^ Gestational age not reported in 4 studies for FPG and 2 for HOMA-IR. ^3^ Weight not reported in 6 studies for FPG and only 1 for HOMA-IR. Mean age for the supplement-based interventions was 30.5 yrs. Overweight and normal-weight pregnancies were defined as pre-pregnancy BMI ≥ 25 or BMI < 25, respectively. If pre-pregnancy weight was unavailable, overweight and normal-weight pregnancies were defined as BMI ≥ 30 or BMI < 30, respectively. Significant *p*-values are expressed in bold (*p ≤* 0.05).

**Table 5:** Subgroup analysis of dietary vs. control interventions.

Category	Outcome Measure	RCTs (n)	MD	95% CI	*p* -Value	I^2^
Fasting Plasma Glucose (FPG, mmol/L)						
**Main analysis**	*Overall*	10	−0.17	(−0.35, 0.01)	0.06	89
**Maternal Age**	*<Mean age*	7	−0.26	(−0.50, −0.03)	**0.03**	91
	*≥Mean age*	3	0.05	(−0.29, 0.81)	0.79	78
**Gestational Age ^1^**	*<28 weeks*	5	−0.25	(−0.51, 0.01)	0.06	86
	*≥28 weeks*	4	−0.08	(−0.33, 0.16)	0.51	88
**Weight** **(pre-pregnancy)** **(kg/m^2^)**	*Normal weight (<25)*	3	−0.32	(−0.74, 0.10)	0.14	88
	*Overweight (≥25)*	7	−0.11	(−0.34, 0.12)	0.35	89
**Diagnostic Criteria for GDM ^2^**	*ADA*	4	−0.51	(−0.78, −0.24)	**0.0003**	69
	*Other*	5	−0.02	(−0.21, 0.17)	0.83	88
**Geographic Region**	*Western* **country**	5	0.02	(−0.13, 0.16)	0.83	63
	*Non-western country*	5	−0.41	(−0.66, −0.15)	**0.002**	85
**Study Duration ^3^**	*Acute*	2	0.19	(−0.25, 0.63)	0.39	82
	*Longitudinal*	7	−0.29	(−0.49, −0.08)	**0.006**	88
Postprandial Glucose (PPG, mmol/L)						
**Main analysis**	*Overall*	5	−0.23	(−0.69, 0.24)	0.34	95
**Maternal Age**	*<Mean age*	4	−0.32	(−0.97, 0.32)	0.33	95
	*≥Mean age*	1	−0.14	(−0.30, 0.02)	0.10	NA
**Gestational Age ^1^**	*<28 weeks*	2	0.18	(−0.44, 0.81)	0.57	98
	*≥28 weeks*	2	−0.24	(−0.68, 0.20)	0.29	79
**Weight** **(pre-pregnancy)** **(kg/m^2^)**	*Normal weight (<25)*	2	−0.24	(−0.68, 0.20	0.29	79
	*Overweight (≥25)*	3	−0.25	(−0.92, 0.42)	0.46	97
**Diagnostic Criteria for GDM**	*ADA*	1	−2.5	(−3.81, −1.19)	**0.0007**	NA
	*Other*	4	−0.02	(−0.46, 0.42)	0.93	96
**Geographic Region**	*Western* **country**	2	0.18	(−0.44, 0.81)	0.57	98
	*Non-western country*	3	−0.63	(−1.33, 0.06)	0.07	88
**Study Duration**	*Acute*	1	0.50	(0.39, 0.61)	**<0.0001**	NA
	*Longitudinal*	4	−0.36	(−0.73, 0.02)	**0.06**	82
Glycated haemoglobin (HbA_1c_, %)						
**Main analysis**	*Overall*	4	−0.08	(−0.23, 0.08)	0.34	70
**Maternal Age**	*<Mean age*	3	−0.11	(−0.34, 0.12)	0.33	80
	*≥Mean age*	1	0.00	(−0.20, 0.20)	1	NA
**Gestational Age ^1^**	*<28 weeks*	1	−0.20	(−0.64, 0.24)	0.356	NA
	*≥28 weeks*	2	−0.03	(−0.21, 0.15)	0.71	0
**Weight** **(pre-pregnancy)** **(kg/m^2^)**	*Normal weight (<25)*	2	0.03	(−0.03, 0.09)	0.35	0
	*Overweight (≥25)*	2	−0.24	(−0.40, −0.08)	**0.003**	0
**Diagnostic Criteria for GDM ^2^**	*ADA*	1	−0.25	(−0.42, −0.07)	**0.007**	NA
	*Other*	2	0.03	(−0.03, 0.09)	0.35	0
**Geographic Region**	Western country	2	−0.03	(−0.21, 0.15)	0.71	0
	Non-western country	2	−0.10	(−0.37, 0.18)	0.48	89
HOMA-IR						
**Main analysis**	*Overall*	5	−1.15	(−2.12, −0.17)	**0.02**	94
**Maternal Age**	*<Mean age*	3	−1.94	(−2.33, −1.56)	**<0.0001**	0
	*≥Mean age*	2	−0.06	(−0.30, 0.19)	0.66	0
**Gestational Age**	*<28 weeks*	1	−1.9	(−2.36, −1.44)	**<0.0001**	NA
	*≥28 weeks*	4	−0.91	(−1.84, 0.02)	0.05	90
**Weight** **(pre-pregnancy)** **(kg/m^2^)**	*Normal weight (<25)*	2	−1.00	(−2.86, 0.86)	0.29	93
	*Overweight (≥25)*	3	−1.27	(−2.77, 0.22)	0.10	94
**Diagnostic Criteria for GDM**	*ADA*	2	−1.92	(−2.33, −1.51)	**<0.0001**	0
	*Other*	3	−0.54	(−1.39, 0.31)	0.22	87
**Geographic Region**	*Western country*	3	−0.54	(−1.39, 0.31)	0.22	87
	*Non-western country*	2	−1.92	(−2.33, −1.51)	**<0.0001**	0
**Study Duration**	*Acute*	1	0.10	(−0.42, 0.62)	0.699	NA
	*Longitudinal*	4	−1.48	(−2.71, −0.26)	**0.02**	95

^1^ Gestational age not reported in 1 study for FPG, PPG and HbA_1c_. ^2^ Diagnostic criteria for GDM not reported in 1 study for FPG and HbA_1c_. ^3^ Study duration not reported in 1 study for FPG. Mean age for the supplement-based interventions was 30.6 yrs. Overweight and normal-weight pregnancies were defined as pre-pregnancy BMI ≥ 25 or BMI < 25, respectively. If pre-pregnancy weight was unavailable, overweight and normal-weight pregnancies were defined as BMI ≥ 30 or BMI < 30, respectively. Significant *p*-values are expressed in bold (*p ≤* 0.05).

**Table 6:** Subgroup analysis of exercise vs. control interventions.

Category	Outcome Measure	RCTs (n)	MD	95% CI	*p* -Value	I^2^
Fasting Plasma Glucose (FPG, mmol/L)						
**Main analysis**	*Overall*	5	−0.10	(−0.20, −0.01)	**0.04**	0
**Maternal Age**	*<Mean age*	4	−0.15	(−0.27, −0.04)	**0.01**	0
	*≥Mean age*	1	0.00	(−0.17, 0.17)	1.00	NA
**Gestational Age ^1^**	*<28 weeks*	1	−0.12	(−0.35, 0.11)	0.336	NA
	*≥28 weeks*	3	−0.16	(−0.29, −0.03)	**0.02**	0
**Weight** **(pre-pregnancy)** **(kg/m^2^)**	*Normal weight (<25)*	3	−0.16	(−0.29, −0.03)	**0.02**	0
	*Overweight (≥25)*	2	−0.04	(−0.18, 0.10)	0.56	0
**Diagnostic Criteria for GDM**	*75 g OGTT*	2	−0.08	(−0.24, 0.09)	0.37	40
	*Other*	3	−0.12	(−0.16, −0.07)	0.17	36
Postprandial Glucose (PPG, mmol/L)						
**Main analysis**	*Overall*	4	−0.24	(−0.59, 0.12)	0.17	82
**Maternal Age**	*<Mean age*	3	−0.39	(−0.71, −0.07)	**0.02**	70
	*≥Mean age*	1	0.20	(−0.08, 0.48)	0.161	NA
**Gestational Age ^1^**	*<28 weeks*	1	−0.64	(−0.94, −0.34)	**0.0002**	NA
	*≥28 weeks*	2	−0.21	(−0.39, −0.03)	**0.02**	0
**Weight** **(pre-pregnancy)** **(kg/m^2^)**	*Normal weight (<25)*	2	−0.21	(−0.39, −0.03)	**0.02**	0
	*Overweight (≥25)*	2	−0.22	(−1.04, 0.60)	0.60	94
**Diagnostic Criteria for GDM**	*75 g OGTT*	2	0.00	(−0.38, 0.37)	0.98	79
	*Other*	2	−0.58	(−0.83, −0.32)	**<0.0001**	0
Glycated haemoglobin (HbA_1c_, %)						
**Main analysis**	*Overall*	3	0.04	(−0.19, 0.27)	0.73	56
**Maternal Age**	*<Mean age*	1	−0.10	(−0.32, 0.12)	0.377	NA
	*≥Mean age*	2	0.38	(−0.56, 1.31)	0.43	50
**Weight** **(pre-pregnancy) ^2^** **(kg/m^2^)**	*Normal weight (<25)*	1	0.1	(−0.03, 0.23)	0.12	NA
	*Overweight (≥25)*	1	−0.10	(−0.32, 0.12)	0.377	NA
**Geographic Region**	*Western country*	2	0.02	(−0.17, 0.21)	0.83	59
	*Non-western country*	1	1.2	(−0.32, 2.72)	0.130	NA

^1^ Gestational age not reported in 1 study for FPG and PPG. ^2^ Weight not reported in 1 study for HbA_1c_. Mean age for the supplement-based interventions was 33.1 yrs. Overweight and normal-weight pregnancies were defined as pre-pregnancy BMI ≥ 25 or BMI < 25, respectively. If pre-pregnancy weight was unavailable, overweight and normal-weight pregnancies were defined as BMI ≥ 30 or BMI < 30, respectively. Significant *p*-values are expressed in bold (*p ≤* 0.05).

## Data Availability

Not applicable.

## Supplementary Materials

The following supporting information can be downloaded at: https://www.mdpi.com/article/10.3390/nu15020323/s1; Table S1: Predetermined search strategy, Table S2: Risk of bias assessment, Table S3: GRADE Assessment for nutritional supplement-based intervention, Table S4: GRADE Assessment for diet-based intervention, Table S5: GRADE Assessment for exercise-based intervention, Figure S1: Funnel plot of fasting plasma glucose (mmol/L) in nutritional supplement interventions, Figure S2: Funnel plot of HOMA-IR in nutritional supplement interventions, Figure S3: Funnel plot of fasting plasma glucose (mmol/L) in dietary interventions, Figure S4: Funnel plot of HOMA-IR in dietary interventions, Figure S5: Funnel plot of fasting plasma glucose (mmol/L) in exercise interventions.
