# Supplementary material for: Nutritional and Exercise-Focused Lifestyle Interventions and Glycemic Control in Women with Diabetes in Pregnancy: A Systematic Review and Meta-Analysis of Randomized Clinical Trials
Source: Nutrients. 2023 Jan 9;15(2):323. doi: 10.3390/nu15020323 (PMC9864154; doi:10.3390/nu15020323)
Supplement: Supplementary file 1 [file nutrients-15-00323-s001.zip › Table S1.pdf]

Table S1. Predetermined search strategy

SEARCH CATEGORIES	USED SEARCH TERMS
1. POPULATION	(diabet*) OR (GDM) OR (IDDM) OR (NIDDM) OR (MODY) OR (LADA)
2. INTERVENTION	(exercise) OR (sports) OR (activity) OR (fitness) OR (training) OR (accelerom*) OR (pedomet*) OR (steps) OR (weightlifting)
3. OUTCOME	(insulin) OR (glucose) OR (glycaemic*) OR (glycemic*) OR (blood sugar) OR (glycated haemoglobin) OR (glycated haemoglobin) OR (HbA1c) OR (OGTT) OR (AUC) OR (HOMA)
4. STUDY TYPE	(randomized controlled trial) OR (random*) OR (placebo*) OR (single blin*) OR (double blind*) OR (triple blind*) OR (clinical trial) AND (humans NOT animals)
5. COMBINED SEARCH	(1) AND (2) AND (3) AND (4)
