# Supplementary material for: Nutritional and Exercise-Focused Lifestyle Interventions and Glycemic Control in Women with Diabetes in Pregnancy: A Systematic Review and Meta-Analysis of Randomized Clinical Trials
Source: Nutrients. 2023 Jan 9;15(2):323. doi: 10.3390/nu15020323 (PMC9864154; doi:10.3390/nu15020323)
Supplement: Supplementary file 1 [file nutrients-15-00323-s001.zip › Table S2.pdf]

Table S2. Risk of bias assessment

Study ID	Experimental	Comparator	Outcome	Weight	D1	D2	D3	D4	D5	Overall
Asemi (2013)	DASH diet	Standard care diet	FG, PPG, HbA1c	NA	+	+	+	+	+	+
Aslfalah (2020)	ALA supplement	Cellulose acetate	FG, HbA1c	NA	+	+	+	+	+	+
Bo (2014)	Brisk walks 20 min/day	Standard care diet	FG, PPG, HbA1c	NA	+	+	+	+	+	+
Brankston (2004)	Resistence exercise	Standard care diet	FG, PPG	NA	+	+	+	!	+	!
de Barros (2010)	Resistance exercise	Standard antenatal care	FG	NA	+	+	+	!	+	!
Fei (2014)	SBOS supplement	Standard antenatal care	FG, HOMA-IR	NA	+	!	+	!	+	!
Grant (2011)	Low GI diet	Standard care diet	FG, PPG	NA	+	+	+	!	+	!
Hajimoosayi (2020)	Ginger supplement	Placebo supplement	FG, PPG, HOMA	NA	+	+	+	!	+	!
Halse (2014)	Home-based exercises	Standard antenatal care	FG, PPG, HbA1c	NA	+	+	+	!	+	!
Hernandez (2014)	Higher complex CHO	Standard care diet	FG, PPG	NA	+	+	+	!	+	!
Hernandez (2016)	Higher complex CHO	Standard care diet	HOMA-IR	NA	+	+	+	!	+	!
Jamilian (2015)	Soy diet	Control diet	FG, HOMA-IR	NA	+	+	+	+	+	+
Jamilian (2018)	Fish oil supplement	Placebo supplement	FG, HOMA-IR	NA	+	+	+	+	+	+
Jamilian (2019)	Mg-zinc-calcium-Vit D	Placebo supplement	FG	NA	+	+	+	+	+	+
Jamilian (2020)	Flaxseed oil/ ALA supplement	Placebo supplement	FG, HOMA-IR	NA	+	+	+	+	+	+
Kokic (2018)	Exercise programme / walks	Standard antenatal care	FG, PPG	NA	+	+	+	+	+	+
Lindsay (2015)	Probiotic (Lactobacillus salivarius)	Placebo supplement	FG, HOMA	NA	+	+	+	+	+	+
Louie (2011)	Low glycemic index (target GI~50)	Standard care diet	HOMA, HbA1c	NA	+	+	+	+	+	+
Ma (2015)	Intensive low glycemic load	Standard care diet	FG, PPG, HbA1c	NA	+	+	+	!	+	!
Ostadmohammadi (2019)	Zinc Gluconate / Vit E supplement	Placebo supplement	FG, HOMA	NA	+	+	+	+	+	+
Perichart-Perera (2012)	Low glycemic index	Standard care diet	FG	NA	+	+	+	!	+	!
Qazi (2020)	Moderate intensity aerobics	Standard antenatal care	HbA1c	NA	+	+	+	!	+	!
Rae (2000)	Moderately energy restricted diet	Standard care diet (not resticted)	FG, HbA1c	NA	+	+	+	+	+	+
Rasmussen (2020)	Low CHO morning intake	High CHO morning intake	FG, HOMA	NA	+	+	+	!	+	!
Valentini (2012)	"Ethnic meal plan"	Standard care diet	FG, PPG, HbA1c	NA	!	+	+	!	-	-
Wang (2015)	Oil-rich diet	Standard care diet	FG, PPG	NA	!	+	+	!	+	!
Yao (2015)	DASH diet	Standard care diet	FG, HOMA	NA	!	+	+	!	+	!

+	Low risk
!	Some concerns
-	High risk
D1	Randomisation process
D2	Deviations from interventions
D3	Missing outcome data
D4	Measurement of the outcome
D5	Selection of the reported result
