# Supplementary material for: Nutritional and Exercise-Focused Lifestyle Interventions and Glycemic Control in Women with Diabetes in Pregnancy: A Systematic Review and Meta-Analysis of Randomized Clinical Trials
Source: Nutrients. 2023 Jan 9;15(2):323. doi: 10.3390/nu15020323 (PMC9864154; doi:10.3390/nu15020323)
Supplement: Supplementary file 1 [file nutrients-15-00323-s001.zip › Table S3.pdf]

Table S3. GRADE Assessment for nutritional supplement-based intervention

Outcome	№ of studies	№ of participants		Certainty assessment					Effect estimate	Grade
		Diet-based interventions	Placebo or standard care	Risk of bias	Inconsistency	Indirectness	Imprecision	Other considerations		
Fasting glucose (mmol/L)	8	264	268	Not serious	Serious [a]	Serious [b]	Not serious	None	**-0.3** [-0.55 , -0.06]	⊕⊕◯◯ Low
Postprandial glucose (mmol/L)	1	37	33	Not serious	Not serious	Not serious	Very serious [c]	Publication bias strongly suspected [d]	**-0.1** [-0.66 , 0.46]	⊕◯◯◯ Very low
HbA1c (%)	1	30	30	Not serious	Not serious	Not serious	Not serious	Publication bias strongly suspected [d]	**-0.15** [-0.22 , 0.08]	⊕⊕⊕◯ Moderate
HOMA-IR	6	204	208	Not serious	Not serious	Serious [b]	Not serious	None	**-0.04** [-0.58 , -0.22]	⊕⊕⊕◯ Moderate

a. Due to high unexplained heterogeneity.
b. Due to substantial differences in interventions and comparisons.
c. The 95% CI included benefits and harms.
d. Only reported by 1 study.
