# Supplementary material for: Nutritional and Exercise-Focused Lifestyle Interventions and Glycemic Control in Women with Diabetes in Pregnancy: A Systematic Review and Meta-Analysis of Randomized Clinical Trials
Source: Nutrients. 2023 Jan 9;15(2):323. doi: 10.3390/nu15020323 (PMC9864154; doi:10.3390/nu15020323)
Supplement: Supplementary file 1 [file nutrients-15-00323-s001.zip › Table S4.pdf]

Table S4. GRADE Assessment for diet-based intervention

Outcome	№ of studies	№ of participants		Certainty assessment					Effect estimate	Grade
		Diet-based interventions	Placebo or standard care	Risk of bias	Inconsistency	Indirectness	Imprecision	Other considerations		
Fasting glucose (mmol/L)	10	309	296	Serious [a]	Serious [b]	Not serious	Not serious	None	**-0.17** [-0.35 , 0.01]	⊕⊕◯◯ Low
Postprandial glucose (mmol/L)	5	125	133	Not serious	Serious [b]	Not serious	Very serious [c]	None	**-0.23** [-0.69 , 0.24]	⊕◯◯◯ Very low
HbA1c (%)	4	167	158	Not serious	Serious [b]	Serious [d]	Serious [c]	None	**-0.08** [-0.23 , 0.08]	⊕◯◯◯ Very low
HOMA-IR	5	107	107	Not serious	Serious [b]	Not serious	Not serious	None	**-1.15** [-2.12 , 0.17]	⊕⊕⊕◯ Moderate

a. Problems with allocation concealment and blinding of participants/researchers/outcome assessors.
b. Due to high unexplained heterogeneity.
c. The 95% CI included benefits and harms.
d. Due to substantial differences in interventions.
