# Supplementary material for: Nutritional and Exercise-Focused Lifestyle Interventions and Glycemic Control in Women with Diabetes in Pregnancy: A Systematic Review and Meta-Analysis of Randomized Clinical Trials
Source: Nutrients. 2023 Jan 9;15(2):323. doi: 10.3390/nu15020323 (PMC9864154; doi:10.3390/nu15020323)
Supplement: Supplementary file 1 [file nutrients-15-00323-s001.zip › Table S5.pdf]

Table S5. GRADE Assessment for exercise-based intervention

Outcome	№ of studies	№ of participants		Certainty assessment					Effect estimate	Grade
		Diet-based interventions	Placebo or standard care	Risk of bias	Inconsistency	Indirectness	Imprecision	Other considerations		
Fasting glucose (mmol/L)	5	181	185	Not serious	Not serious	Serious [a]	Not serious	None	**-0.1** [-0.20 , -0.01]	⊕⊕⊕◯ Moderate
Postprandial glucose (mmol/L)	4	149	153	Not serious	Serious [b]	Serious [a]	Serious [c]	None	**-0.24** [-0.59 , 0.11]	⊕◯◯◯ Very low
HbA1c (%)	3	144	146	Not serious	Serious [b]	Serious [a]	Serious [c]	None	**0.04** [-0.19 , 0.27]	⊕◯◯◯ Very low
HOMA-IR	1	99	101	Not serious	Not serious	Not serious	Serious [c]	Publication bias strongly suspected [d]	**0.00** [-0.88 , 0.88]	⊕⊕◯◯ Low

a. Due to substantial differences in interventions and comparisons.
b. Due to high unexplained heterogeneity.
c. The 95% CI included benefits and harms.
d. Only reported by 1 study.
