# Supplementary figures and images for: Nutritional and Exercise-Focused Lifestyle Interventions and Glycemic Control in Women with Diabetes in Pregnancy: A Systematic Review and Meta-Analysis of Randomized Clinical Trials
Source: Nutrients. 2023 Jan 9;15(2):323. doi: 10.3390/nu15020323 (PMC9864154; doi:10.3390/nu15020323)

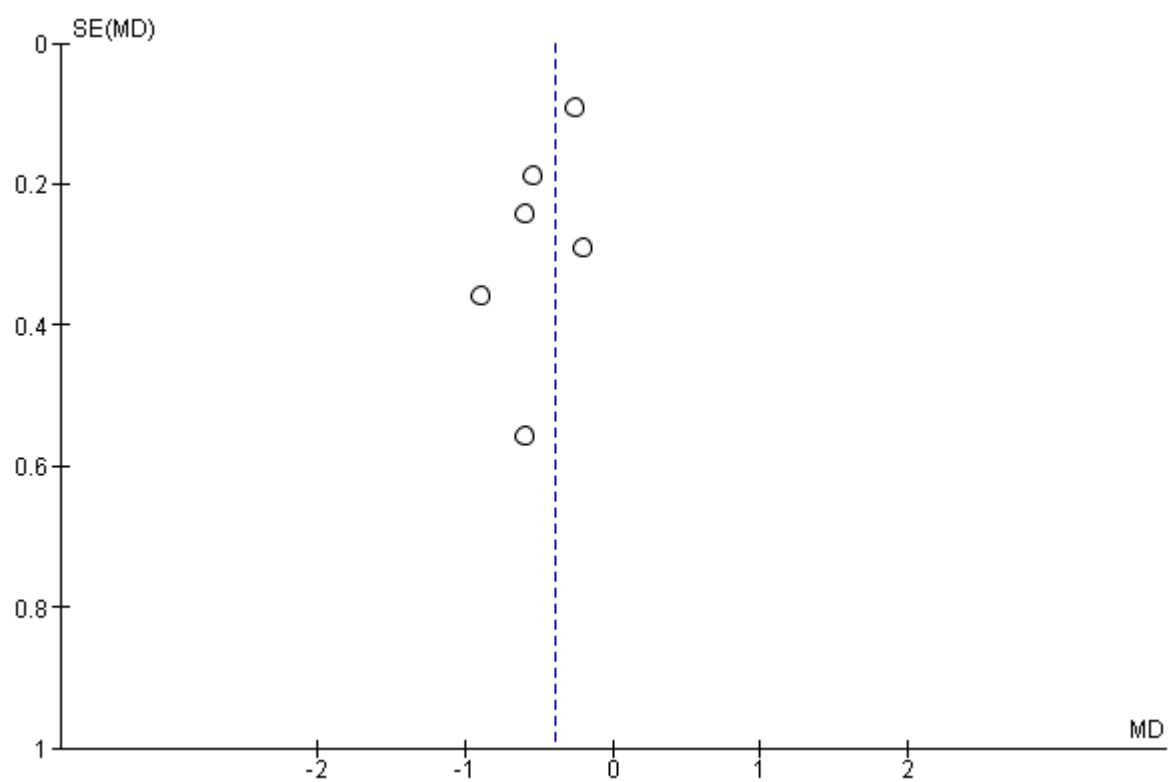

**Figure S2.** Funnel plot of HOMA-IR in nutritional supplement interventions

Supplement: Supplementary file 1 [file nutrients-15-00323-s001.zip › Figure S2.pdf]

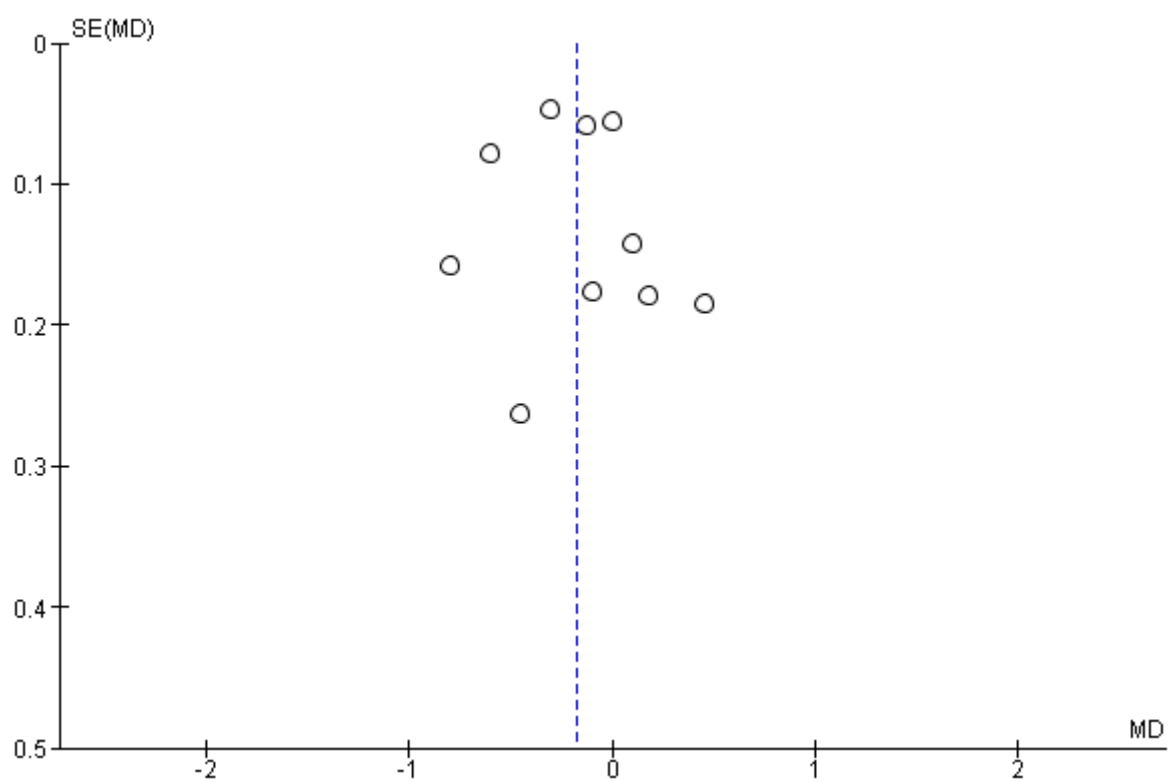

**Figure S3.** Funnel plot of fasting plasma glucose (mmol/L) in dietary interventions

Supplement: Supplementary file 1 [file nutrients-15-00323-s001.zip › Figure S3.pdf]

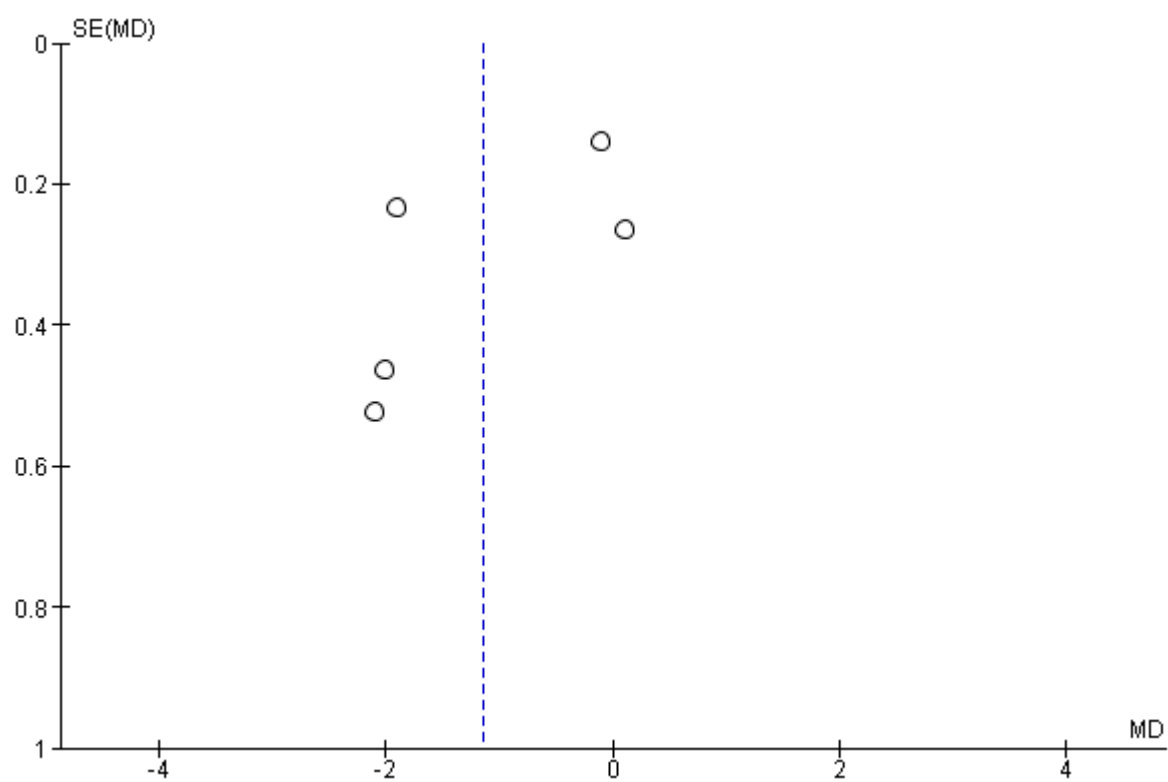

**Figure S4.** Funnel plot of HOMA-IR in dietary interventions

Supplement: Supplementary file 1 [file nutrients-15-00323-s001.zip › Figure S4.pdf]

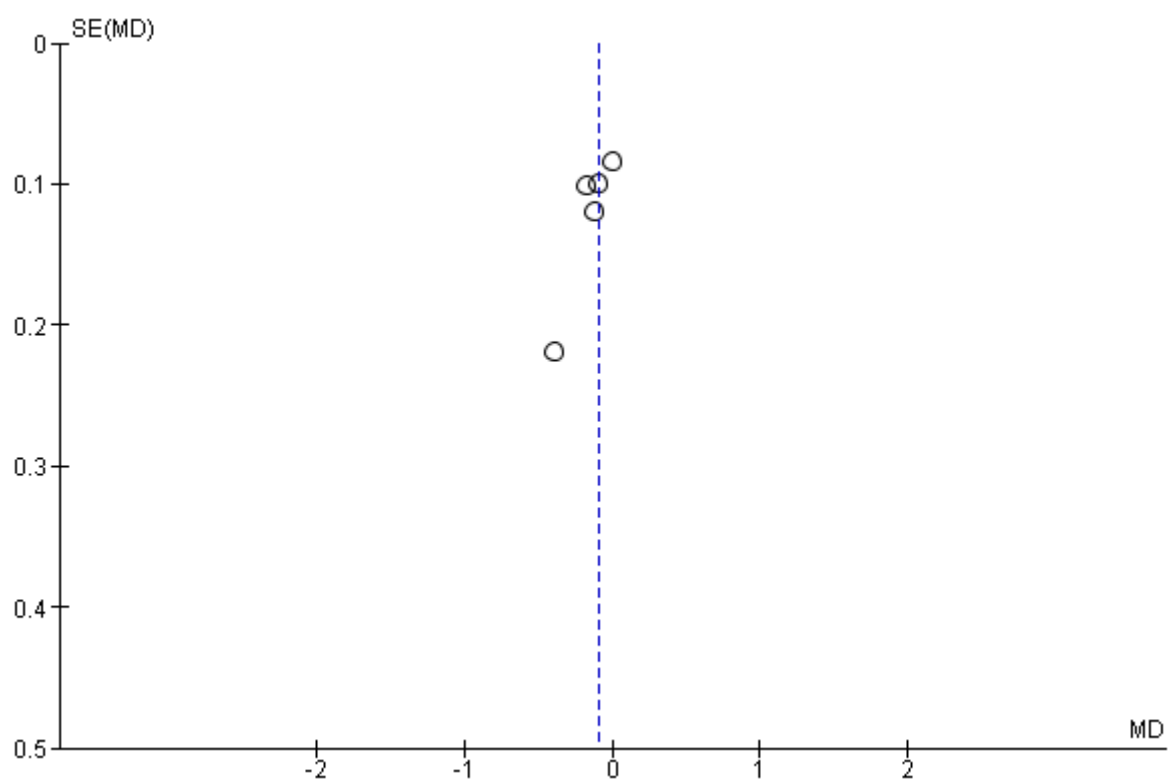

**Figure S5.** Funnel plot of fasting plasma glucose (mmol/L) in exercise interventions

Supplement: Supplementary file 1 [file nutrients-15-00323-s001.zip › Figure S5.pdf]
